# Original Versus Generic Eltrombopag in Patients with Immune Thrombocytopenia: A Prospective Multi-Center Experience on Efficacy and Safety

**DOI:** 10.3390/jcm15020634

**Published:** 2026-01-13

**Authors:** Serhat Çelik, Zeynep Tuğba Karabulut, Cem Selim, Rafiye Çiftçiler, Abdulkerim Yıldız, Samet Yaman, İbrahim Ethem Pınar, Ayşe Hilal Eroğlu Küçükdiler, Nuray Gül Açar, Aysun Şentürk Yıkılmaz, Vehbi Demircan, Dilek Keskin, İbrahim Halil Açar, Ekin Kırcalı, Meltem Kurt Yüksel

**Affiliations:** 1 Department of Hematology, Yenimahalle Training and Research Hospital, Yıldırım Beyazıt University, Ankara 06370, Türkiye; 2 Department of Hematology, Faculty of Medicine, Ankara University, Ankara 06620, Türkiye; meltemkurt@hotmail.com; 3 Department of Hematology, Adana City Traning and Research Hospital, Adana 01370, Türkiye; drztkarabulutguven@gmail.com; 4 Department of Hematology, Mehmet Akif İnan Traning and Research Hospital, Şanlıurfa 63040, Türkiye; drcemselim@gmail.com; 5 Department of Hematology, Faculty of Medicine, Selçuk University, Konya 42090, Türkiye; rafiyesarigul@gmail.com; 6 Department of Hematology, Çorum Erol Olçok Traning and Research Hospital, Hitit University, Çorum 19040, Türkiye; akerim@hotmail.com (A.Y.); drsametyaman@hotmail.com (S.Y.); 7 Department of Hematology, Isparta City Hospital, Isparta 32200, Türkiye; dr.ethem@hotmail.com; 8 Department of Hematology, Faculty of Medicine, Adnan Menderes University, Aydın 09100, Türkiye; e_hilal@hotmail.com; 9 Department of Hematology, Faculty of Medicine, Çukurova University, Adana 01330, Türkiye; nuraygulacar@gmail.com; 10 Department of Hematology, Denizli State Hospital, Denizli 20010, Türkiye; senturkaysun@gmail.com; 11 Department of Hematology, Faculty of Medicine, Dicle University, Diyarbakır 21280, Türkiye; vehbi_dmrcn@hotmail.com; 12 Department of Hematology, Kanuni Sultan Süleyman Traning and Research Hospital, Istanbul 34303, Türkiye; dilekkeskin84@hotmail.com; 13 Department of Hematology, Osmaniye State Hospital, Osmaniye 80000, Türkiye; halil_acar_63@hotmail.com; 14 Department of Hematology, Ankara Medicana International Hospital, Ankara 06510, Türkiye; ekinkircali@gmail.com

**Keywords:** drug formulation, eltrombopag, fatigue, generic drugs, purpura, thrombocytopenic, idiopathic, thrombopoietin receptor agonist

## Abstract

**Background/Objectives**: Eltrombopag, a thrombopoietin receptor agonist, is widely used in the treatment of relapsed or refractory (R/R) immune thrombocytopenia (ITP). This study aimed to compare the efficacy, safety, and tolerability of generic eltrombopag (Rompag^®^) with original eltrombopag (Revolade^®^) in adult patients with R/R ITP. **Methods**: In this prospective, multicenter study conducted at 10 centers, 104 adult ITP patients were followed for at least 3 months. A total of 35 (33.7%) patients received Rompag^®^ and 69 (66.3%) received Revolade^®^. The primary endpoint was platelet (PLT) response, defined as achieving a PLT count ≥50 × 10^9^/L and at least a twofold increase from baseline, without the need for rescue therapy or transfusion. Secondary endpoints included bleeding rates, fatigue-related quality of life, adverse events (AEs), and rescue therapy requirements. **Results**: PLT response was achieved in 94.2% of patients in the Revolade^®^ group and 85.7% in the Rompag^®^ group (*p* = 0.16). Bleeding rates decreased significantly in both groups (Revolade^®^: 56.5% to 2.9%, *p* < 0.001; Rompag^®^: 62.9% to 2.9%, *p* < 0.001). Although overall AE rates were similar (30.4% in the Revolade^®^ group and 42.9% in the Rompag^®^ group; *p* = 0.22), arthralgia (28.6% vs. 7.2%, *p* = 0.01) and vomiting (11.4% vs. 0%, *p* = 0.008) were more frequent with Rompag^®^. **Conclusions**: Both generic and original eltrombopag demonstrated no statistically significant difference in efficacy in achieving PLT response, reducing bleeding, and improving fatigue-related quality of life in adult patients with R/R ITP. Although minor differences in AE profiles were observed, particularly arthralgia and vomiting, both formulations showed acceptable safety and tolerability.

## 1. Introduction

Immune thrombocytopenia (ITP) is characterized by immune-mediated destruction of platelets, primarily within the reticuloendothelial system, driven by autoantibodies and other dysregulated immune mechanisms. This process leads to both increased platelet clearance and insufficient platelet production [1]. First-line treatment typically involves corticosteroids, such as prednisone or dexamethasone and/or intravenous immunoglobulin (IVIG) [2]. Although the initial response rate to corticosteroids is high, approximately 80% of patients require second-line treatment [3].

A wide range of second-line treatment options are available; however, there is a paucity of randomized controlled trials that directly compare these options [1]. The American Society of Hematology’s ITP clinical guidelines recommend rituximab as a second-line treatment alternative to splenectomy and conditionally recommend thrombopoietin receptor agonists as an alternative to rituximab [4].

Eltrombopag is a small-molecule, non-peptide thrombopoietin receptor agonist that promotes megakaryocyte proliferation and platelet production [2]. The efficacy and safety of eltrombopag in patients with ITP were demonstrated in multiple randomized and open-label studies [5,6,7,8]. With the expiration of the patent for the branded formulation Revolade^®^, generic versions of eltrombopag such as Rompag^®^ have become available. Generic drugs are approved primarily based on bioequivalence studies rather than on real-world effectiveness or safety data. However, differences in excipients or manufacturing processes may influence tolerability and side-effect profiles, which may not be captured in bioequivalence testing. Although the efficacy and safety of original eltrombopag have been extensively studied in clinical trials and in the real world, there is a lack of comparative data evaluating generic alternatives. There is no study that has systematically compared the efficacy and safety of the generic and original forms of eltrombopag in adult patients with ITP.

In this multicenter prospective study, the objective was to compare the efficacy, safety, and tolerability of generic eltrombopag (Rompag^®^) with that of original eltrombopag (Revolade^®^) in adult patients diagnosed with ITP.

## 2. Materials and Methods

### 2.1. Study Design and Population

A total of 104 adult patients with relapsed or refractory ITP were enrolled in this prospective multicenter study conducted across 10 hematology centers between January 2024 and January 2025. The aim of the study was to compare the efficacy and safety of the original eltrombopag (Revolade^®^, Novartis Pharma AG, Basel, Switzerland) with its generic counterpart (Rompag^®^, Abdi İbrahim İlaç Sanayi ve Ticaret A.Ş., Istanbul, Türkiye). Adult patients (≥18 years) with a primary diagnosis of ITP refractory or relapsed and initiated on eltrombopag (Revolade^®^ or Rompag^®^) were included in the study. Patients were required to have at least 3 months of follow-up data. Exclusion criteria included active malignancy, secondary thrombocytopenia, eating disorders, prior exposure to eltrombopag, or concomitant use of other thrombopoietin receptor agonists. Additionally, patients who had received any ITP therapy other than corticosteroids or IVIG within 6 months prior to eltrombopag initiation were excluded. Patients received either Revolade^®^ or Rompag^®^ as per physician discretion. The starting dose and subsequent dose adjustments were made according to national ITP guidelines and clinical judgment.

This study was conducted in accordance with the Declaration of Helsinki and was approved by the Clinical Research Ethics Committee of Yıldırım Beyazıt University Yenimahalle Training and Research Hospital (Date of Approval: 20 December 2023; Protocol No: E-2023-70). Written informed consent was obtained from all participants.

### 2.2. Data Collection and Endpoints

Baseline characteristics such as age, gender, comorbidities, and ITP characteristics such as previous treatments and bleeding symptoms were recorded. The classification of toxicity and adverse events (AEs) was conducted in accordance with the Common Terminology Criteria for Adverse Events, version 5.0 [9]. The assessment of fatigue-related quality of life was conducted utilizing the Functional Assessment of Chronic Illness Therapy–Fatigue (FACIT-Fatigue) scale, a tool that has been validated for use in a range of hematological and autoimmune disorders [6,10]. The FACIT-Fatigue questionnaire was administered face-to-face under the supervision of the attending physician at baseline, at week 2, and at subsequent monthly visits.

The primary endpoint was PLT response, defined as achieving a PLT count ≥50 × 10^9^/L and at least a twofold increase from baseline at any follow-up timepoint, without the need for rescue treatment or blood transfusion [6,11]. Secondary endpoints included AEs, bleeding episodes, requirement for rescue therapy, and fatigue-related quality of life measured by the FACIT-Fatigue scale. Data were collected at baseline, Week 2, Month 1, Month 2, and Month 3 of eltrombopag. Patients received either Revolade^®^ or Rompag^®^ as per physician discretion. The starting dose and subsequent dose adjustments were made according to national ITP guidelines and clinical judgment. Rescue therapy was defined as the administration of any additional therapy required to control bleeding or to increase PLT counts in cases of insufficient response. Patients were monitored regularly for endpoints.

### 2.3. Statistical Analysis

The Kolmogorov–Smirnov test was used to assess the distribution of continuous variables. Parametric variables were expressed as mean ± standard deviation and compared using Student’s *t*-test. Non-parametric variables were expressed as median (range) or median (interquartile range, IQR) and compared using the Mann–Whitney U test. Categorical variables were summarized as frequencies and percentages and compared using chi-square or Fisher’s exact test, as appropriate.

A pre-specified formal sample size calculation was not performed; instead, all eligible patients treated during the study period were included. Given the real-world, multicenter nature of the study, the analysis was designed to be descriptive and exploratory rather than powered for hypothesis testing. Between-group estimates are presented with p-values and 95% confidence intervals to convey the precision of comparisons in lieu of a formal power calculation.

For within-group comparisons of repeated measures over time (e.g., PLT count, and FACIT-Fatigue scores), the Friedman test—a non-parametric alternative to repeated-measures ANOVA—was applied. When significant overall differences were detected, pairwise comparisons between timepoints were conducted using the Wilcoxon signed-rank test. Changes in bleeding symptoms were analyzed using Cochran’s Q test to evaluate differences across multiple timepoints within each treatment group. Pairwise comparisons for binary outcomes between specific timepoints were performed using McNemar’s test. The data were analyzed using the Statistical Package for the Social Sciences (SPSS) for Windows (release 25.0; SPSS Inc., Chicago, IL, USA). A two-tailed p-value of less than 0.05 was considered statistically significant in all tests. declared.

## 3. Results

### 3.1. Descriptive Characteristics of the Study Population

A total of 104 adult patients with ITP were included in the study, of whom 69 (66.3%) received original eltrombopag (Revolade^®^) and 35 (33.7%) received generic eltrombopag (Rompag^®^). The median follow-up in our cohort was 4.1 months (IQR, 3.0–6.7). The median age was 53 (range: 19–69) years in the Revolade group and 46 (range: 19–69) years in the Rompag group (*p* = 0.31). The proportion of female patients was 62.3% (*n* = 43) in the Revolade group and 54.3% (*n* = 19) in the Rompag group (*p* = 0.43). The median PLT count at treatment initiation was 11 × 10^9^/L (range: 2–30) in the Revolade group and 9 × 10^9^/L (range: 1–28) in the Rompag group (*p* = 0.09). The rate of patients who had undergone splenectomy prior to eltrombopag treatment was 8.7% (*n* = 8) in the Revolade group and 14.3% (*n* = 5) in the Rompag group (*p* = 0.5). The median number of prior ITP treatments was two in both groups (IQR: 2–7 for Revolade, 2–6 for Rompag) (*p* = 0.1). Bleeding symptoms were present in 61% (*n* = 42) of patients in the Revolade group and 71% (*n* = 25) in the Rompag group (*p* = 0.5). Baseline demographics, ITP-related stratification variables, comorbidities, and bleeding characteristics did not differ significantly between the two groups (*p* > 0.05 for all, Table 1), although some numerical differences were observed.

### 3.2. Primary Endpoint and Platelet Response Rate

**Figure 1:**
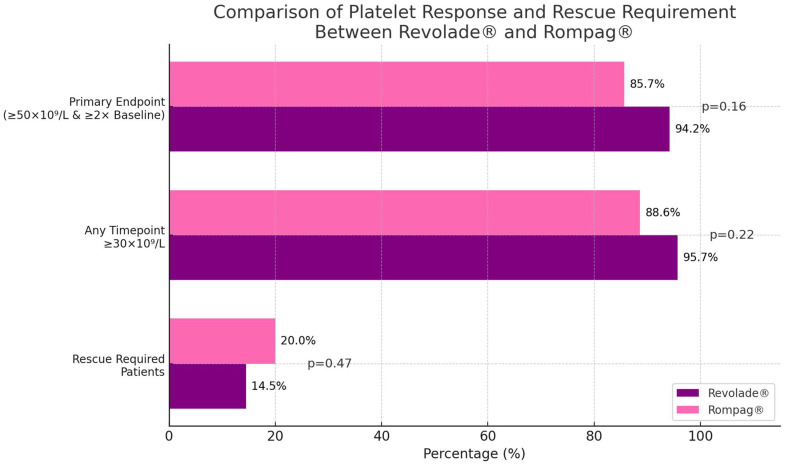
Comparison of platelet response and rescue requirement between the Revolade^®^ and Rompag^®^ groups.

The primary endpoint was PLT response, defined as achieving a PLT count ≥50 × 10^9^/L and at least a twofold increase from baseline, without the need for rescue treatment or transfusion. The platelet response rate was 94.2% in patients treated with Revolade^®^ and 85.7% in those treated with Rompag^®^, corresponding to an absolute difference of 8.5% (95% CI, −4.3% to +21.3%; *p* = 0.16). At any timepoint during follow-up, a platelet count of ≥30 × 10^9^/L was achieved in 95.7% of patients (*n* = 66) in the Revolade^®^ group and 88.6% (*n* = 31) in the Rompag^®^ group (*p* = 0.22) (Figure 1).

**Figure 2:**
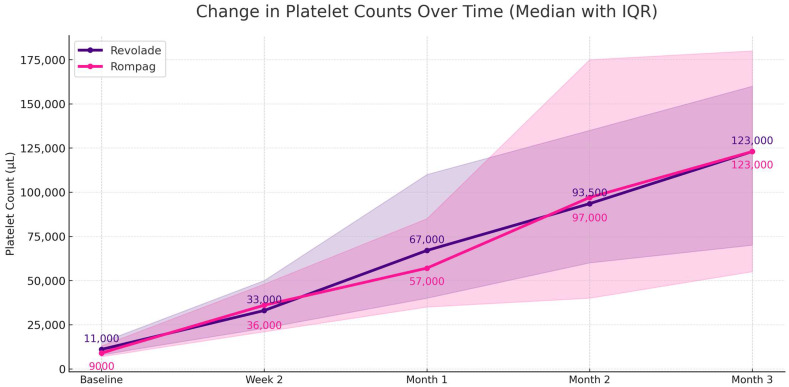
Change in platelet counts over time (median with interquartile range) between the Revolade^®^ and Rompag^®^ groups.

The median PLT values over time showed a consistent upward trend in both treatment groups. In the Revolade^®^ group, the median PLT at baseline, week 2, month 1, month 2, and month 3 were 11 × 10^9^/L (IQR: 7–18), 33 × 10^9^/L (IQR: 19–57), 67 × 10^9^/L (IQR: 43–109), 93.5 × 10^9^/L (IQR: 49.5–140.1), and 123 × 10^9^/L (IQR: 68–158), respectively. In the Rompag^®^ group, these values were 9 × 10^9^/L (IQR: 4–12), 36 × 10^9^/L (IQR: 21–47.5), 57 × 10^9^/L (IQR: 24–85), 97 × 10^9^/L (IQR: 39.5–164), and 122 × 10^9^/L (IQR: 55–187.5), respectively (Figure 2, Supplementary Table S2). A statistically significant overall change in PLT counts across these timepoints was observed in both groups (Revolade: χ^2^ = 142.01, *p* < 0.001; Rompag: χ^2^ = 96.42, *p* < 0.001). In both cohorts, the most pronounced increase occurred within the first two weeks, followed by a gradual yet statistically significant rise through month 3 (Supplementary Table S3). Pairwise Wilcoxon signed-rank tests confirmed that the increases from baseline to each subsequent timepoint up to month 3 were statistically significant in both groups (*p* < 0.05 for all; Supplementary Table S3).

### 3.3. Bleeding Symptoms

**Figure 3:**
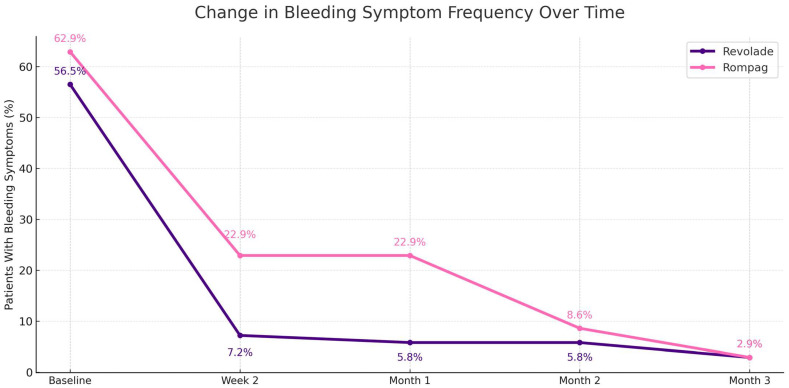
Changes in the frequency of bleeding symptoms over time between Revolade^®^ and Rompag^®^ groups.

In the Revolade^®^ group, the proportion of patients who experienced at least one bleeding event was 56.5% (*n* = 39) at baseline, 7.2% (*n* = 5) at week 2, 5.8% (*n* = 4) at month 1, 5.8% (*n* = 4) at month 2, and 2.9% (*n* = 2) at month 3. None of the bleeding events were life-threatening. In the Rompag group, these rates were 62.9% (*n* = 22), 22.9% (*n* = 8), 22.9% (*n* = 8), 8.6% (*n* = 3), and 2.9% (*n* = 1), respectively (Figure 3). Bleeding symptoms decreased over time in both groups: Revolade^®^ group, χ^2^(4) = 124.85, *p* < 0.001, and Rompag^®^ group, χ^2^(4) = 47.08, *p* < 0.001. The bleeding events over time in both groups is shown in Supplementary Table S4.

### 3.4. FACIT-Fatigue Score

**Figure 4:**
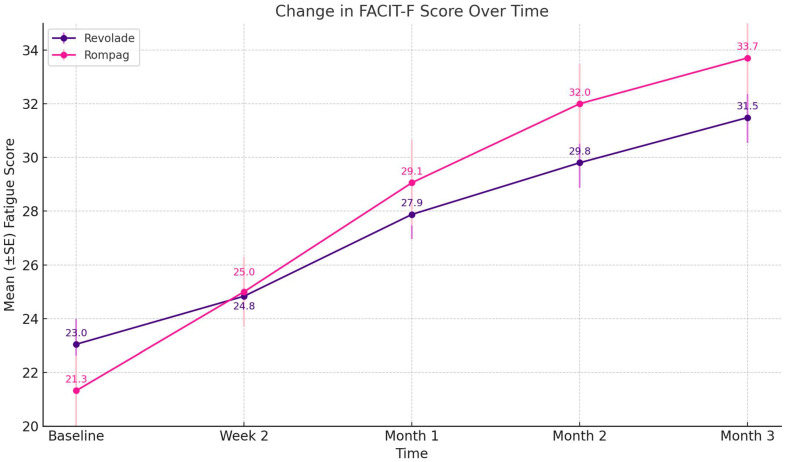
Changes in the FACIT-Fatigue score over time between Revolade^®^ and Rompag^®^ groups.

A significant improvement in FACIT fatigue score was observed over the course of treatment in both groups (Revolade: χ^2^ = 122.40, *p* < 0.001; Rompag: χ^2^ = 108.44, *p* < 0.001). In the Revolade group, the mean fatigue score increased from 23.0 (range: 7–39) at baseline to 31.5 (range: 12–50) at month 3 (Figure 4). Similarly, in the Rompag group, the score rose from 21.3 (range: 7–39) at baseline to 33.7 (range: 14–50) at month 3 (Supplementary Table S5). When pairwise comparisons between timepoints were evaluated, a statistically significant reduction in fatigue symptoms was observed in all comparisons within both groups (*p* < 0.001) (Supplementary Table S6).

### 3.5. Adverse Events

Adverse events (AEs) were reported in 30.4% (*n* = 21) of patients in the Revolade^®^ group and 42.9% (*n* = 15) in the Rompag^®^ group (*p* = 0.21). The most frequently observed AEs were headache in 17.3% of patients (*n* = 18), arthralgia in 14.4% (*n* = 15), and extremity pain in 9.6% (*n* = 10). Most AEs were manageable and predominantly grade 1 in severity. In the Revolade^®^ group, a single patient experienced a grade 2 elevation in liver transaminases, while no grade ≥3 events were observed. In contrast, the Rompag^®^ group had one patient with grade 2 and one with grade 3 transaminase elevation, two patients with grade 2 arthralgia, and one patient with grade 2 extremity pain. Treatment discontinuation due to adverse events occurred in one patient from the Rompag^®^ group (grade 3 hepatotoxicity). Overall, both treatments were generally well tolerated (Table 2). A statistically significant difference was observed between the two groups in terms of arthralgia and nausea. Arthralgia was reported in 28.6% (*n* = 10) of patients receiving Rompag^®^, compared to 7.2% (*n* = 5) in the Revolade^®^ group (*p* = 0.01). While no cases of vomiting were recorded in the Revolade^®^ group, it was observed in 11.4% (*n* = 4) of patients in the Rompag^®^ group (*p* = 0.008).

### 3.6. Rescue Therapy

During the study period, rescue therapy was required in 17 patients (16.3%) (Supplementary Table S7). This included 10 patients (14.5%) in the Revolade^®^ group and 7 patients (20%) in the Rompag^®^ group (*p* = 0.47). Among the rescue therapies, only 3 patients (17.6%) underwent splenectomy. The most commonly preferred rescue treatments were rituximab in 7 patients (41.2%), corticosteroids in 6 patients (35.3%), and IVIG in 5 patients (29.4%).

## 4. Discussion

This prospective, multicenter study is the first to systematically compare the efficacy and safety of generic eltrombopag (Rompag^®^) with original eltrombopag (Revolade^®^) in adult patients with relapsed or refractory immune ITP. The findings of the present study demonstrate that both formulations provide effective thrombopoietic responses, significant reductions in bleeding symptoms, and improvements in fatigue-related quality of life, without significant differences in the primary efficacy endpoint or serious safety outcomes.

In phase 3, double-blind, placebo-controlled trials, eltrombopag achieved PLT response rates of 59% and 79% in the 6-week and 6-month RAISE studies, respectively [6,12]. In a randomized placebo-controlled phase 3 trial conducted in China, the response rate was reported as 57.7% [13]. Long-term and real-world studies such as EXTEND and CITE have reported even higher response rates, ranging from 81% to 94% [8,11,14,15,16,17,18,19,20]. In our study, the PLT response rate was 94.2% in patients treated with original eltrombopag (Revolade^®^) and 85.7% in those treated with generic eltrombopag (Rompag^®^).

Platelet response following eltrombopag treatment has been reported to begin within the first week and become more pronounced by the second week [6,8,12,17,19,20]. In the study by Tomiyama et al., a lower dose of 12.5 mg also resulted in a marked response by the second week, with sustained effect observed from the fourth week onward [7]. In the phase 4 multicenter prospective CITE study, the platelet response was reported to become prominent by the first month [11]. In the final analysis of the EXTEND study, more than half of the patients maintained a consistent response for at least 25 weeks [14]. In our study, consistent with previous reports, a notable platelet response was observed by the second week in both groups and was sustained throughout the three-month follow-up period.

One of the important goals in the treatment of ITP is the prevention of bleeding symptoms. In the study by Bussel et al., the bleeding rate decreased from 61% to 39% over a 6-week period following eltrombopag treatment; in the placebo group, this rate decreased from 79% to 60% [12]. In the RAISE study, the bleeding rate in patients treated with eltrombopag decreased from 79% to 33% [6]. In the EXTEND study, which had a longer follow-up period, the bleeding rate decreased from 57% to 16% at the end of one year of follow-up [14]. In our study, consistent with the previous literature, a significant decrease in bleeding symptoms was observed in both the Revolade^®^ and Rompag^®^ groups; the bleeding rate decreased to 2.9% in both groups. The low number of patients with severe bleeding in our study may have contributed to the more pronounced reduction in bleeding observed after eltrombopag compared to other studies.

Fatigue is a commonly observed symptom in ITP, although its underlying mechanisms remain unclear [21,22]. Despite its prevalence, fatigue is often underestimated by clinicians, yet it significantly impairs patients’ quality of life [23]. According to the ITP World Impact Survey, 50% of patients (*n* = 1507) reported experiencing fatigue both at the time of diagnosis and five years later [24]. The FACIT-Fatigue scale, a validated instrument commonly used in ITP clinical trials, was employed in our study to evaluate fatigue. In the RAISE study, although improvements in FACIT-Fatigue scores were observed in the eltrombopag arm, the difference between groups did not reach statistical significance [6]. In contrast, the CITE study demonstrated a significant improvement in FACIT-Fatigue scores following eltrombopag treatment [11]. Similarly, in our study, both groups showed a significant and sustained improvement in fatigue scores over the three-month follow-up period.

AE rates associated with eltrombopag have been reported to range between 21.8% and 92% across previous studies, with the majority being grade 1–2 in severity [7,8,11,12,13,14,15,18,20,25,26]. Overall, eltrombopag has been considered well tolerated. In our study, eltrombopag was also well tolerated, with AE rates observed at 30.4% in the Revolade^®^ group and 42.9% in the Rompag^®^ group. In a meta-analysis including 2765 patients with ITP treated with eltrombopag, the most commonly reported AEs were nasopharyngitis (19.3%), headache (11.7%), nausea (9.6%), diarrhea (9.2%), and elevated ALT levels (6.9%) [27]. In our cohort, the most frequent AE was headache (17.3%). Similarly, headache was also the most commonly reported AE in the RAISE trial (30%), EXTEND trial (28%), and REPEAT study (21%) [6,8,14,25]. Although AE rates were numerically higher in the Rompag^®^ group in our study, a statistically significant difference was observed only for arthralgia and nausea. This difference may be attributed to formulation-related factors such as excipients or bioavailability variations between the generic and original products, or to chance variation due to the limited sample size. Therefore, clinicians should be aware that generic and brand formulations, while pharmacologically equivalent, may differ in the frequency of uncommon adverse reactions.

One of the key challenges following eltrombopag treatment is the need for rescue therapy. In the EXTEND study, 28% of patients required rescue interventions; however, this study had a relatively long median follow-up period of approximately 100 weeks. In the RAISE trial, the rate was reported as 18%, and two real-world studies reported rates of 14% and 16% [6,20,26]. In our study, there was no statistically significant difference between the Revolade^®^ and Rompag^®^ groups in terms of rescue therapy requirements, with rates of 14.5% and 20%, respectively.

### Limitations

First, as an observational study, there is a potential for residual bias or confounding, despite efforts to standardize baseline characteristics. Second, eltrombopag has known dietary restrictions: patients are advised to avoid dietary fat and divalent cations (e.g., calcium and magnesium) within a 4–6 h window around dosing to ensure optimal absorption. While this is an important aspect of patient management, it is challenging to achieve standardized monitoring of dietary adherence across multiple centers. Nevertheless, the CITE study reported a 96% adherence rate to dietary and dosing instructions [11]. Third, the follow-up period in our study was relatively short, as it was designed to compare the original and generic formulations, which limited the ability to assess long-term efficacy and adverse events. Fourth, because the study included all eligible patients without a priori size determination and the group sizes were unequal (69 vs. 35), it may be underpowered to detect small between-group differences. Post hoc, assuming true response rates around 90% and using the actual sample sizes, the minimal detectable absolute difference at 80% power and a two-sided α of 0.05 would be approximately 17–18 percentage points; the observed difference of 8.5 percentage points therefore lies below this threshold. Consequently, non-significant results should be interpreted with caution.

## 5. Conclusions

This prospective, multicenter, real-world data indicated that both generic and original eltrombopag demonstrated similar efficacy in achieving PLT response, reducing bleeding symptoms, and improving fatigue-related quality of life in adult patients with relapsed or refractory ITP. Although minor differences were observed in AE profiles, particularly in terms of arthralgia and vomiting, the overall safety and tolerability of both formulations were acceptable. The efficacy and safety profiles of both original and generic eltrombopag should be further evaluated in larger, population-based studies with extended follow-up periods to better assess long-term outcomes.

## Figures and Tables

**Table 1:** Descriptive Characteristics of the Study Groups.

Variable	Revolade (*n* = 69)	Rompag (*n* = 35)	*p*
Median age (years)	53 (19–69)	46 (19–69)	0.31
Sex (female) (*n*, %)	43 (62.3)	19 (54.3)	0.43
Stratification variables			
Median platelet count (×10^9^/L)	11 (2–30)	9 (1–28)	0.09
Platelet level ≤ 10 × 10^9^/L (*n*, %)	34, 49.3	23, 65.7	0.17
Splenectomy (*n*, %)	6, 8.7	5, 14.3	0.5
Number of previous cITP treatments (median)	2 (2–7)	2 (2–6)	0.1
Number of previous cITP treatments (>2)	18, 26.1	10, 28.5	0.8
Comorbidities			
Diabetes mellitus (*n*, %)	7, 10.1	3, 8.6	0.8
Essential hypertension (*n*, %)	20, 29	9, 25.9	0.3
Smokers (*n*, %)	12, 17.4	9, 25.9	0.3
Bleeding symptoms (*n*, %)	42, 60.9	25, 71.4	0.3
Clinically significant bleeding symptoms (*n*, %)	26, 37.7	19, 54.3	0.11

cITP: chronic immune thrombocytopenia.

**Table 2:** Comparison of Adverse Event Frequencies Between Rompag and Revolade Groups.

Variable	Revolade (*n* = 69)	Rompag (*n* = 35)	*p*
Increased ALT concentration (*n*, %)			
Baseline	2, 2.9	1, 2.9	0.99
Month 1	4, 5.8	2, 5.7 (1 grade 2)	0.3
Month 2	5, 7.2 (1 grade 2)	3, 8.6 (1 grade 2, 1 grade 3)	0.46
Month 3	5, 7.2	1, 2.9	0.35
Increased creatinine concentration (*n*, %)	1, 1.5	-	0.99
Headache (*n*, %)	11, 15.9	7, 20	0.81
Upper respiratory tract infection (*n*, %)	1, 1.5	1, 2.9	1
Nausea (*n*, %)	4, 5.8	5, 14.3	0.15
Vomiting (*n*, %)	-	4, 11.4	**0.01**
Arthralgia (*n*, %)	5, 7.2	10, 28.6 (2 grade 2)	**0.008**
Back pain (*n*, %)	3, 4.3	6, 17.1	0.28
Insomnia (*n*, %)	5, 7.2	3, 8.6	0.8
Extremity pain (*n*, %)	4, 5.8	5, 14.3 (1 grade 2)	0.12
Cataract (*n*, %)	-	1, 2.9	0.34
Thromboembolic events (*n*, %)	2, 2.9	-	0.55
Any grade 3 or 4 adverse event (*n*, %)	-	1, 2.9	0.34

ALT: alanine aminotransferase. Bolded values indicate *p*-values < 0.05.

## Data Availability

The data presented in this study are available on reasonable request from the corresponding author. The data are not publicly available due to privacy and ethical restrictions.

## Supplementary Materials

The following supporting information can be downloaded at: https://www.mdpi.com/article/10.3390/jcm15020634/s1, Table S1: Previous ITP treatments and time since last therapy in patients receiving Revolade^®^ or Rompag^®^. Table S2: Median and interquartile range of platelet counts over time, Table S3: Wilcoxon signed-rank test p-values for pairwise comparisons of platelet counts between sequential timepoints in Revolade^®^ and Rompag^®^ groups, Table S4: McNemar test results for bleeding frequency between timepoints, Table S5: FACIT-Fatigue scores by group and timepoint, Table S6: Pairwise comparisons of FACIT-Fatigue scores by timepoint, Table S7: Patients requiring rescue therapy after eltrombopag treatment.

## Abbreviations

The following abbreviations are used in this manuscript:

ITP	Immune Thrombocytopenia
PLT	Platelet
TPO-RA	Thrombopoietin Receptor Agonist
AE	Adverse Event
FACIT-F	Functional Assessment of Chronic Illness Therapy–Fatigue
IQR	Interquartile range
SD	Standard deviation
CTCAE	Common Terminology Criteria for Adverse Events
SPSS	Statistical Package for the Social Sciences
