# Supplementary material for: Original Versus Generic Eltrombopag in Patients with Immune Thrombocytopenia: A Prospective Multi-Center Experience on Efficacy and Safety
Source: J Clin Med. 2026 Jan 13;15(2):634. doi: 10.3390/jcm15020634 (PMC12841940; doi:10.3390/jcm15020634)
Supplement: Supplementary file 1 [file jcm-15-00634-s001.zip › jcm-3941033-supplementary.pdf]

Supplementary Table S1. Previous ITP treatments and time since last therapy in patients receiving Revolade® or Rompag®.

Previous treatment	Revolade® n (%)	Rompag® n (%)	Median interval since last administration (months)
Corticosteroids	69, (100)	35, (100)	-
IVIG	69, (100)	35, (100)	2 (0.5-4)
Rituximab	14, (20.3)	11, 31.4	9 (6–15)
Splenectomy	6, (8.7)	5, (14.3)	11 (4-62)
Azathioprine	2, (2.9)	1, (2.9)	10 (6-19)
Danazol	1, (1.5)	-	8
Mycophenolate mofetil	-	1, 2.9	6

IVIG: intravenous immunoglobulin

Supplementary Table S2. Median and Interquartile Range (IQR) of Platelet Counts Over Time

Timepoint	Group	Median (/µL)	Q1 (25%)	Q3 (75%)
Baseline	Revolade	11000	7000	18000
Baseline	Rompag	9000	4000	12000
Week 2	Revolade	33000	19000	57000
Week 2	Rompag	36000	21000	47500
Month 1	Revolade	67000	43000	109000
Month 1	Rompag	57000	24000	85000
Month 2	Revolade	93500	49500	140750
Month 2	Rompag	97000	39500	164000
Month 3	Revolade	123000	68000	158000
Month 3	Rompag	122000	55000	187500

Supplementary Table S3. Wilcoxon signed-rank test p-values for pairwise comparisons of platelet counts between sequential timepoints in Revolade and Rompag groups.

Timepoint Comparison	Revolade (p)	Rompag (p)
Baseline vs Week 2	<0.000001	<0.000001
	$9.6x10^{-12}$	$1.7x10^{-10}$
Week 2 vs Month 1	0.0002	0.0004
Month 1 vs Month 2	0.0017	0.0001
Month 2 vs Month 3	0.0091	0.028

Supplementary Table S4. McNemar Test Results for Bleeding Frequency Between Timepoints

Group	Comparison	χ^2	p-value
Revolade	Baseline vs Week 2	0.000	< .0001
Revolade	Baseline vs Month 1	0.000	< .0001
Revolade	Baseline vs Month 2	0.000	< .0001
Revolade	Baseline vs Month 3	0.000	< .0001
Revolade	Week 2 vs Month 1	0.000	1.0
Revolade	Week 2 vs Month 2	1.000	1.0
Revolade	Week 2 vs Month 3	1.000	0.38
Revolade	Month 1 vs Month 2	1.000	1.0
Revolade	Month 1 vs Month 3	1.000	0.63
Revolade	Month 2 vs Month 3	1.000	0.63
Rompag	Baseline vs Week 2	0.000	0.0001
Rompag	Baseline vs Month 1	1.000	0.0005
Rompag	Baseline vs Month 2	0.000	< .0001
Rompag	Baseline vs Month 3	0.000	< .0001
Rompag	Week 2 vs Month 1	3.000	1.0
Rompag	Week 2 vs Month 2	1.000	0.22
Rompag	Week 2 vs Month 3	0.000	0.03
Rompag	Month 1 vs Month 2	2.000	0.29
Rompag	Month 1 vs Month 3	1.000	0.07
Rompag	Month 2 vs Month 3	0.000	0.5

Supplementary Table S5. FACIT Fatigue Scores by Group and Timepoint

Timepoint	Group	Mean	SD
Baseline	Revolade	23.05	7.66
Week 2	Revolade	24.83	6.82
Month 1	Revolade	27.88	7.49
Month 2	Revolade	29.80	7.55
Month 3	Revolade	31.48	7.65
Baseline	Rompag	21.32	7.60
Week 2	Rompag	25.00	7.63
Month 1	Rompag	29.06	9.30
Month 2	Rompag	32.00	8.68

Month 3	Rompag	33.71	7.86

Supplementary Table S6. Pairwise Comparisons of FACIT Fatigue Scores by Timepoint (Wilcoxon Test)

Group	Comparison	Wilcoxon Statistic	p-value
Rompag	Baseline vs Month 3	2.50	< .0001
Rompag	Week 2 vs Month 2	6.00	< .0001
Rompag	Baseline vs Month 2	13.00	< .0001
Revolade	Week 2 vs Month 2	108.50	< .0001
Revolade	Week 2 vs Month 3	144.00	< .0001
Revolade	Baseline vs Month 3	160.50	< .0001
Revolade	Baseline vs Month 2	173.50	< .0001
Revolade	Week 2 vs Month 1	74.50	< .0001
Rompag	Week 2 vs Month 3	1.00	< .0001
Revolade	Month 1 vs Month 3	133.50	< .0001
Rompag	Month 1 vs Month 3	2.00	< .0001
Revolade	Baseline vs Month 1	162.00	< .0001
Rompag	Baseline vs Month 1	8.00	< .0001
Revolade	Month 1 vs Month 2	85.00	< .0001
Revolade	Month 2 vs Month 3	125.50	< .0001
Rompag	Week 2 vs Month 1	0.00	< .0001
Rompag	Month 1 vs Month 2	8.00	< .0001
Rompag	Baseline vs Week 2	6.00	< .0001
Rompag	Month 2 vs Month 3	22.00	0.0004
Revolade	Baseline vs Week 2	120.00	0.0014

Supplementary Table S7. Patients requiring rescue therapy after eltrombopag treatment

Patient number	Type of Eltrombopag	Rescue Therapy
2	Rompag	Corticosteroid, IVIG
3	Revolade	Corticosteroid, IVIG
4	Revolade	Corticosteroid, IVIG

5	Revolade	Corticosteroid, IVIG
7	Rompag	IVIG
22	Revolade	Rituximab
30	Revolade	Rituximab, followed by Mycophenolate Mofetil
42	Revolade	Rituximab
43	Revolade	Plasmapheresis
56	Revolade	Splenectomy
67	Rompag	Corticosteroid
70	Rompag	Corticosteroid
73	Rompag	Splenectomy
79	Revolade	Rituximab
80	Rompag	Rituximab, Romiplostim
84	Rompag	Rituximab
95	Revolade	Splenectomy
104	Revolade	Rituximab–Vincristine

IVIG = Intravenous Immunoglobulin
